# Supplementary material for: PANCDetect: Early Detection of Pancreatic Cancer from Multimodal EHR data with LLM Embeddings
Source: medRxiv. 2025 Nov 14:2025.10.03.25337210. Preprint. [Version 3] doi: 10.1101/2025.10.03.25337210 (PMC12633566; doi:10.1101/2025.10.03.25337210)
Supplement: Supplement 1 [file media-1.pdf]

Supplementary Appendix

Integrated gradient (IG)[1] implementation details:

The IG along the ith dimension is defined as the following:

$$IG_i(x) := (x_i - x_i') \times \int_0^1 \frac{\partial F(x' + \alpha(x - x'))}{\partial x_i} d\alpha$$

where x is the actual input, x' is the baseline (often chosen as zeros or some "neutral" input), F is the prediction function (e.g., the model's risk score for pancreatic cancer), α is an interpolation parameter that smoothly moves from baseline (α=0) to actual input (α=1).

For implementation, the integral can't be computed analytically for deep models, we approximate the integration via the summation with steps m=30 as the following:

$$IG_i^{approx}(x) := (x_i - x_i') \times \sum_{k=1}^{m} \frac{\partial F(x' + \frac{k}{m}(x - x'))}{\partial x_i} \times \frac{1}{m}$$

So practically, IG is "average gradient × input difference."

Supplementary Fig. 1: Age and sex distributions of PANC patients (left) and matched non-PANC controls (right) in the MarketScan cohort. A 1:5 case–control matching strategy based on sex, age, and geographic location ensured consistent demographic ratios between cases and controls. We kept the same matching strategy for UMPH and OneFlorida+ cohorts too.

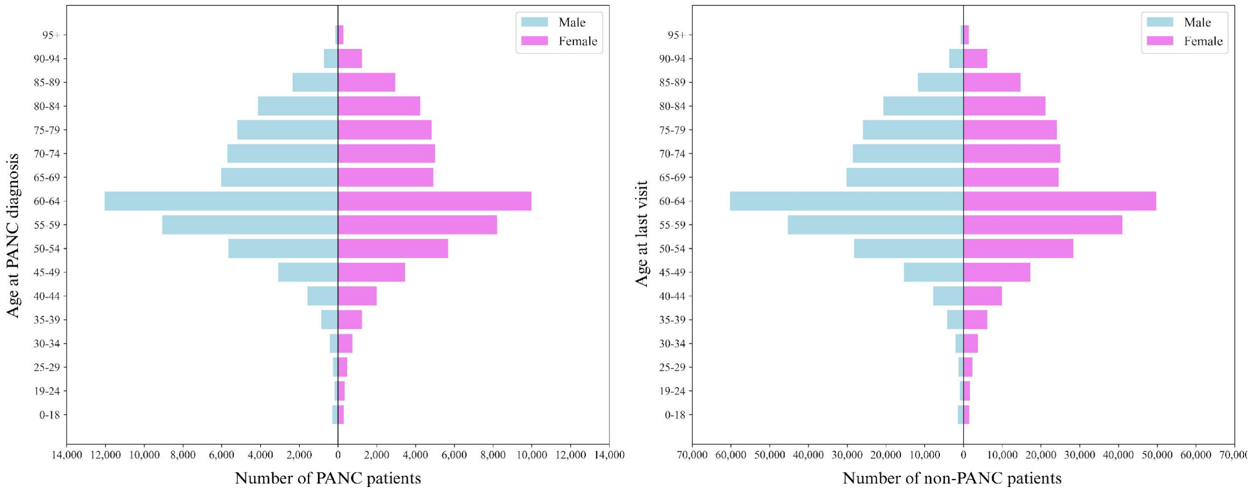

Supplementary Fig. 2: Comparative performance of PANCDetect and CancerRiskNet under the same prediction interval setting. PANCDetect achieved higher AUROC (A) and AUPRG (B) scores across all intervals, with particularly strong gains in long-term predictions (36- and 60-month). CancerRiskNet showed lower discrimination and precision–recall gain, especially at extended horizons. Shaded areas represent 95% confidence intervals.

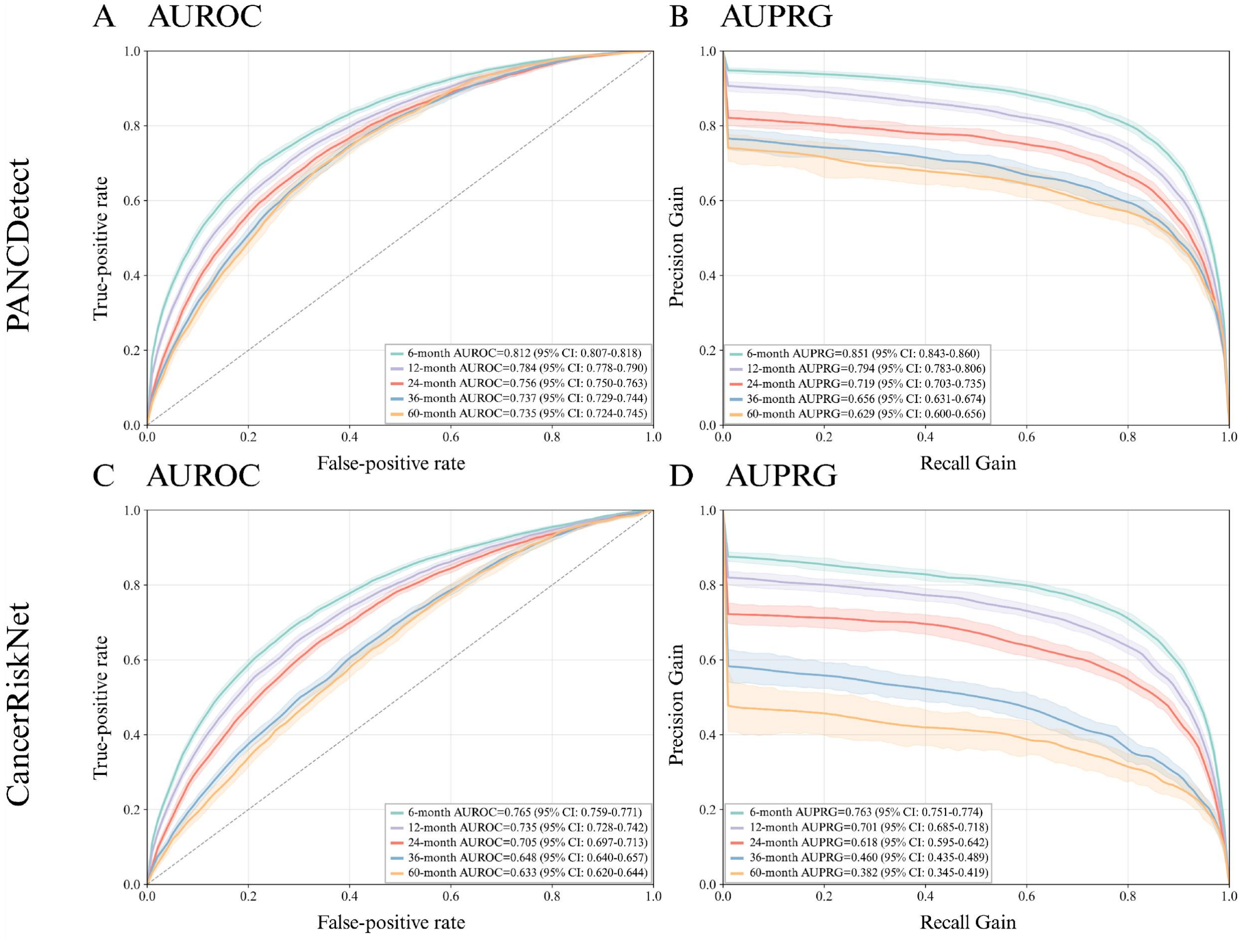

Supplementary Table 1: ICD 9/10 Code for pancreatic cancer

ICD 9 Code	ICD 10 Code
157: Malignant neoplasm of pancreas	C25: Malignant neoplasm of pancreas
157.0: Malignant neoplasm of head of pancreas	C25.0: Malignant neoplasm of head of pancreas
157.1: Malignant neoplasm of body of pancreas	C25.1: Malignant neoplasm of body of pancreas
157.2: Malignant neoplasm of tail of pancreas	C25.2: Malignant neoplasm of tail of pancreas
157.3: Malignant neoplasm of pancreatic duct	C25.3: Malignant neoplasm of pancreatic duct
157.4: Malignant neoplasm of islets of langerhans	C25.4: Malignant neoplasm of endocrine pancreas
157.8: Malignant neoplasm of other specified sites of pancreas	C25.7: Malignant neoplasm of other parts of pancreas
157.9: Malignant neoplasm of pancreas, part unspecified	C25.8: Malignant neoplasm of overlapping sites of pancreas
	C25.9: Malignant neoplasm of pancreas, unspecified

Supplementary Table 2: Other evaluation metrics performance on MarketScan test set.

	**6-month**	**12-month**	**24-month**	**36-month**	**60-month**
**Precision**	0.626 (95% CI: 0.612-0.638)	0.634 (95% CI: 0.616-0.653)	0.502 (95% CI: 0.484-0.525)	0.575 (95% CI: 0.537-0.607)	0.433 (95% CI: 0.405-0.455)
**Recall**	0.360 (95% CI: 0.350-0.369)	0.235 (95% CI: 0.225-0.246)	0.258 (95% CI: 0.246-0.270)	0.106 (95% CI: 0.097-0.116)	0.306 (95% CI: 0.290-0.324)
**F1 Score**	0.458 (95% CI: 0.445-0.467)	0.343 (95% CI: 0.332-0.357)	0.341 (95% CI: 0.327-0.355)	0.180 (95% CI: 0.165-0.194)	0.359 (95% CI: 0.340-0.377)
**Specificity**	0.955 (95% CI: 0.953-0.957)	0.970 (95% CI: 0.969-0.972)	0.944 (95% CI: 0.941-0.947)	0.982 (95% CI: 0.980-0.984)	0.901 (95% CI: 0.895-0.907)

Supplementary Table 3: Other evaluation metrics performance on UMPH cohort (external validation).

	**6-month**	**12-month**	**24-month**	**36-month**	**60-month**
**Precision**	0.200 (95% CI: 0.189-0.214)	0.212 (95% CI: 0.196-0.233)	0.154 (95% CI: 0.137-0.169)	0.132 (95% CI: 0.116-0.152)	0.063 (95% CI: 0.055-0.071)
**Recall**	0.373 (95% CI: 0.350-0.391)	0.244 (95% CI: 0.224-0.262)	0.225 (95% CI: 0.201-0.245)	0.130 (95% CI: 0.110-0.149)	0.223 (95% CI: 0.197-0.254)
**F1 Score**	0.260 (95% CI: 0.244-0.275)	0.227 (95% CI: 0.211-0.245)	0.183 (95% CI: 0.166-0.199)	0.131 (95% CI: 0.113-0.152)	0.098 (95% CI: 0.086-0.111)
**Specificity**	0.900 (95% CI: 0.897-0.904)	0.948 (95% CI: 0.945-0.950)	0.938 (95% CI: 0.935-0.941)	0.961 (95% CI: 0.959-0.964)	0.875 (95% CI: 0.871-0.879)

Supplementary Table 4: Other evaluation metrics performance on OneFlorida+ cohort (external validation).

	**6-month**	**12-month**	**24-month**	**36-month**	**60-month**
**Precision**	0.482 (95% CI: 0.467-0.493)	0.502 (95% CI: 0.484-0.521)	0.351 (95% CI: 0.331-0.371)	0.447 (95% CI: 0.409-0.480)	0.272 (95% CI: 0.239-0.303)
**Recall**	0.618 (95% CI: 0.600-0.634)	0.448 (95% CI: 0.429-0.464)	0.319 (95% CI: 0.299-0.335)	0.203 (95% CI: 0.185-0.224)	0.170 (95% CI: 0.147-0.196)
**F1 Score**	0.541 (95% CI: 0.527-0.553)	0.473 (95% CI: 0.457-0.488)	0.334 (95% CI: 0.317-0.351)	0.279 (95% CI: 0.253-0.302)	0.209 (95% CI: 0.182-0.234)
**Specificity**	0.891 (95% CI: 0.887-0.896)	0.927 (95% CI: 0.924-0.931)	0.898 (95% CI: 0.894-0.903)	0.955 (95% CI: 0.950-0.958)	0.915 (95% CI: 0.908-0.922)

Supplementary Table 5: Other evaluation metrics performance on UMPH hold-out test cohort (finetuning).

	**6-month**	**12-month**	**24-month**	**36-month**	**60-month**
**Precision**	0.799 (95% CI: 0.736-0.862)	0.809 (95% CI: 0.750-0.872)	0.736 (95% CI: 0.649-0.819)	0.807 (95% CI: 0.695-0.906)	0.673 (95% CI: 0.541-0.780)
**Recall**	0.686 (95% CI: 0.610-0.756)	0.654 (95% CI: 0.583-0.767)	0.565 (95% CI: 0.496-0.641)	0.400 (95% CI: 0.333-0.481)	0.438 (95% CI: 0.337-0.547)
**F1 Score**	0.738 (95% CI: 0.679-0.793)	0.724 (95% CI: 0.664-0.775)	0.639 (95% CI: 0.575-0.700)	0.535 (95% CI: 0.457-0.617)	0.530 (95% CI: 0.417-0.632)
**Specificity**	0.969 (95% CI: 0.958-0.979)	0.967 (95% CI: 0.956-0.980)	0.957 (95% CI: 0.941-0.973)	0.980 (95% CI: 0.968-0.991)	0.957 (95% CI: 0.939-0.973)

Supplementary Table 6: Cohort size settings for model training, validation, and finetuning.

	Case/Control				
**Cohorts**	**6-month**	**12-month**	**24-month**	**36-month**	**60-month**
**MarketScan (Model building)**					
Training	59,106/283,37	50,499/238,61	38,387/175,82	29,022/129,31	16,557/70,00
Validation	7,485/35,324	6,393/29,747	4,796/21,980	3,652/16,140	2,111/8,709
Hold-out testing	7,429/35,381	6,401/29,739	4,832/21,945	3,720/16,073	2144/8,676
**OneFlorida+ (External validation)**					
Testing	3,878/23,743	3,074/18,784	2,297/13,307	1,815/10,051	1,187/6,309
**UMPH (External validation)**					
Testing	1,758/26,326	1,585/27,416	1,334/26,728	1,129/24,829	804/21,252
**UMPH (Finetuning)**					
Training	1,422/6,822	1,262/6,166	1,072/5,192	902/4,416	633/3,146
Validation	180/851	161/768	124/659	112/553	91/381
Testing	156/875	162/767	138/646	115/550	80/393

Supplementary Table 7: Ablation study of different embedding generation methods.

Model name & embedding dimension	6-month prediction		60-month prediction	
	**AUROC**	**AUPRG**	**AUROC**	**AUPRG**
**BioGPT (1024)**	0.801 (95% CI: 0.793-0.805)	0.837 (95% CI: 0.829-0.846)	0.710 (95% CI: 0.698-0.721)	0.578 (95% CI: 0.547-0.607)
**GatorTron (1024)**	0.812 (95% CI: 0.806-0.817)	**0.853** (95% CI: 0.836-0.861)	0.729 (95% CI: 0.718-0.739)	0.623 (95% CI: 0.595-0.651)
**Trained from scratch (128)**	0.772 (95% CI: 0.764-0.777)	0.802 (95% CI: 0.791-0.812)	0.682 (95% CI: 0.670-0.694)	0.520 (95% CI: 0.484-0.553)
**OpenAI text-embedding-3-small (1536)**	**0.812** (95% CI: 0.807-0.818)	0.851 (95% CI: 0.843-0.860)	**0.735** (95% CI: 0.724-0.745)	**0.629** (95% CI: 0.600-0.656)

Supplementary Table 8: Ablation study of different modalities used for training.

**Modalities**	**6-month prediction**		**60-month prediction**	
	**AUROC**	**AUPRG**	**AUROC**	**AUPRG**
**Diagnosis**	0.779 (95% CI: 0.773-0.784)	0.810 (95% CI: 0.799-0.819)	0.708 (95% CI: 0.698-0.719)	0.558 (95% CI: 0.527-0.586)
**Diagnosis + Procedure**	0.802 (95% CI: 0.796-0.807)	0.841 (95% CI: 0.832-0.850)	0.721 (95% CI: 0.711-0.732)	0.603 (95% CI: 0.575-0.627)
**Diagnosis + Drug**	0.801 (95% CI: 0.794-0.806)	0.828 (95% CI: 0.818-0.838)	0.729 (95% CI: 0.718-0.739)	0.618 (95% CI: 0.590-0.643)
**Diagnosis + Procedure + Drug**	**0.812** (95% CI: 0.807-0.818)	**0.851** (95% CI: 0.843-0.860)	**0.735** (95% CI: 0.724-0.745)	**0.629** (95% CI: 0.600-0.656)

Reference

1. Sundararajan, M., Taly, A. & Yan, Q. Axiomatic Attribution for Deep Networks. *In Proc. 34th International Conference on Machine Learning (ICML)* (2017).
